# Animal Models of Retinopathy of Prematurity: Advances and Metabolic Regulators

**DOI:** 10.3390/biomedicines12091937

**Published:** 2024-08-23

**Authors:** Meenakshi Maurya, Chi-Hsiu Liu, Kiran Bora, Neetu Kushwah, Madeline C. Pavlovich, Zhongxiao Wang, Jing Chen

**Affiliations:** Department of Ophthalmology, Boston Children’s Hospital, Harvard Medical School, 300 Longwood Avenue, Boston, MA 02115, USA

**Keywords:** retinopathy of prematurity, animal models, oxygen-induced retinopathy, hypoxia, metabolism, amino acid

## Abstract

Retinopathy of prematurity (ROP) is a primary cause of visual impairment and blindness in premature newborns, characterized by vascular abnormalities in the developing retina, with microvascular alteration, neovascularization, and in the most severe cases retinal detachment. To elucidate the pathophysiology and develop therapeutics for ROP, several pre-clinical experimental models of ROP were developed in different species. Among them, the oxygen-induced retinopathy (OIR) mouse model has gained the most popularity and critically contributed to our current understanding of pathological retinal angiogenesis and the discovery of potential anti-angiogenic therapies. A deeper comprehension of molecular regulators of OIR such as hypoxia-inducible growth factors including vascular endothelial growth factors as primary perpetrators and other new metabolic modulators such as lipids and amino acids influencing pathological retinal angiogenesis is also emerging, indicating possible targets for treatment strategies. This review delves into the historical progressions that gave rise to the modern OIR models with a focus on the mouse model. It also reviews the fundamental principles of OIR, recent advances in its automated assessment, and a selected summary of metabolic investigation enabled by OIR models including amino acid transport and metabolism.

## 1. Introduction

Retinopathy of prematurity (ROP) is a potentially blinding eye disease affecting premature infants and is characterized by aberrant proliferation of retinal blood vessels. During its epidemic in the 1940s, when oxygen therapy became a standard procedure in newborn care to enhance the health of preterm children with underdeveloped pulmonary function, Theodore L. Terry described ROP initially as “retrolental fibroplasias” [1,2]. From then to the early 1950s [3], researchers were intrigued to determine the underlying etiology for the persistent pathology of ROP, until the groundbreaking research by Kate Campbell [4] and later investigations [5,6] established that unrestricted supplementary oxygen therapy was a main cause of increased risk of developing ROP.

Premature babies have partially formed retinal vasculature at birth with the peripheral retina being avascular, in contrast to full-term babies whose retinal blood vessels are nearly fully grown. After premature delivery, the comparatively hyperoxic environment of room air (compared with in utero) delays the development and formation of retinal vessels, which is further hindered by oxygen therapy. Decades of studies on clinical and experimental ROP recognized a biphasic nature of ROP pathology, with an initial period of inadequate development of retinal vessels and a second phase of abnormal vascular proliferation driven by hypoxia [7,8,9]. The initial stage of ROP appears from birth to around 30 to 32 weeks postmenstrual age. As the newborn grows and the metabolic activity of the peripheral avascular retina increases, it causes tissue ischemia and hypoxia [10,11,12,13], which can be further exacerbated by termination of oxygen treatment. Hypoxia induces secretion of pro-angiogenic growth factors, including vascular endothelial growth factor (VEGF) as well as erythropoietin (EPO), which can stimulate uncontrolled retinal vascular growth towards the vitreous at the border between avascular and vascular zones, leading to the second hypoxia-induced proliferative phase of ROP. The second phase of ROP starts about weeks 32 to 34 postmenstrual age. Severe cases of proliferative ROP can result in tractional retinal detachment and blindness [7,8,9]. 

While mild ROP may not need treatment and may resolve spontaneously, babies with advanced ROP often requires laser photocoagulation treatment to slow the disease progress, or more increasingly in the past decade treatment with lower dose (compared to adult dose) anti-VEGF therapies to block the pathological growth of blood vessel [14,15,16,17]. Yet, laser treatment may permanently damage the retina, and anti-VEGF therapies have limitations including recurrence, dosing, and long-term safety concerns [18,19], hence necessitating the need for additional ROP research in experimental models. Findings new ways to prevent the initial phase of peripheral avascular retina is very desirable to promote physiological revascularization and prevent the second neovascular phase. In this regard, the role of metabolic risk factors in ROP is increasingly recognized [20], suggesting the need to investigate metabolic regulators in retinopathy development. In addition, given the chronic nature of ROP and its potentially lifelong impact on vision [21], additional longitudinal and long-term studies are warranted. Details about clinical ROP investigation can be found in many previous reviews [18,22,23,24,25,26], whereas this review is more focused on the development and application of animal models of ROP for research and selected metabolic regulators.

The creation of early animal models of ROP was made possible by the findings of oxygen usage as a major risk factor in the 1950s. These models accurately portrayed the harmful vascular consequences of excess oxygen on the growth of retinal vascular tissue in murine, feline, and canine pups, although some differences exist across species. The 1990s saw the modernization of murine ROP models, which led to the standardization of oxygen exposure protocols and techniques for assessing retinopathy severity in mice and rats [27,28]. In experimental models of oxygen-induced retinopathy (OIR), both ROP phases can be simulated to facilitate research into the cellular and molecular mechanisms underlying vaso-obliteration (VO), physiological revascularization, and pathological neovascularization (NV). Along with the availability of genetically modified mice, the mouse model of ROP significantly advanced our knowledge of the molecular basis of ROP pathogenesis and the advancement of possible treatments.

In felines, canines, and rodents, retinal vessels form and mature postnatally, in contrast to humans, whose retinal vessels fully develop before birth [13,29]. Thus, although these ROP models induced by oxygen accurately mimic important vascular features of ROP, they solely reflect the impact of oxygen and do not take into consideration prematurity, which is challenging to simulate in utero. Prematurity-related variables, such as insulin-like growth factors and essential fatty acids (supplied in the third trimester in utero and lacking after premature birth), are studied with supplemental treatments in the OIR models [30,31]. New mouse models of neonatal hyperglycemia-associated retinopathy [32,33] have also been developed recently, as additional risk factors such as neonatal hyperglycemia, a prevalent issue in extremely preterm infants, were linked to severe ROP [34,35,36,37]. New techniques for OIR severity analysis were assisted by recent developments in artificial intelligence (AI) algorithms. This review focuses mainly on the most widely used mouse and rat ROP models [27,28], and their application that allowed recent advances in understanding the basic mechanisms of pathogenesis, including emerging roles of metabolic regulators such as lipids and amino acids.

## 2. Animal Models of Experimental ROP

OIR animal models have proven to be valuable in investigating the effects of oxygen and factors influenced by oxygen on the ROP pathophysiology. These models were created by subjecting neonatal animals to perpetually high or cycling oxygen conditions, including murine [27,28], piscine (fish) [38], feline [39], and canine [40,41,42]. These OIR models mimic both the first VO phase and the second hypoxia-induced NV phase of ROP and are similar to ROP in humans. Animal models of OIR are extensively employed to investigate the underlying processes and assess the efficacy of novel treatments for ROP and other conditions characterized by abnormal blood vessel growth in the retina. These models offer convenient induction of pathological neo-vessels and the ease of monitoring and quantification. Yet the reproducibility of NV can vary among different species [43] and each features distinct advantages and limitations as summarized in Table 1.

### 2.1. The Mouse Model of Oxygen-Induced Retinopathy

#### 2.1.1. Development of Mouse Retinal Vasculature Occurs Postnatally

The development of retinal vascular structures in mice shares similar morphological and spatial patterns as humans [29,44], yet the temporal development of retinal vasculature and neuron differentiation is much delayed in mice, which is a significant distinction from humans. The retinal vasculature in mice begins to grow after birth, whereas, in a human fetus, it does so relatively early in the second trimester, allowing fully mature retinal vasculature at full-term birth [29,45,46]. Hence, studies of physiological and pathological retinal angiogenesis in mice have taken advantage of their developing retinal vasculature throughout the first few postnatal weeks.

**Figure 1:**
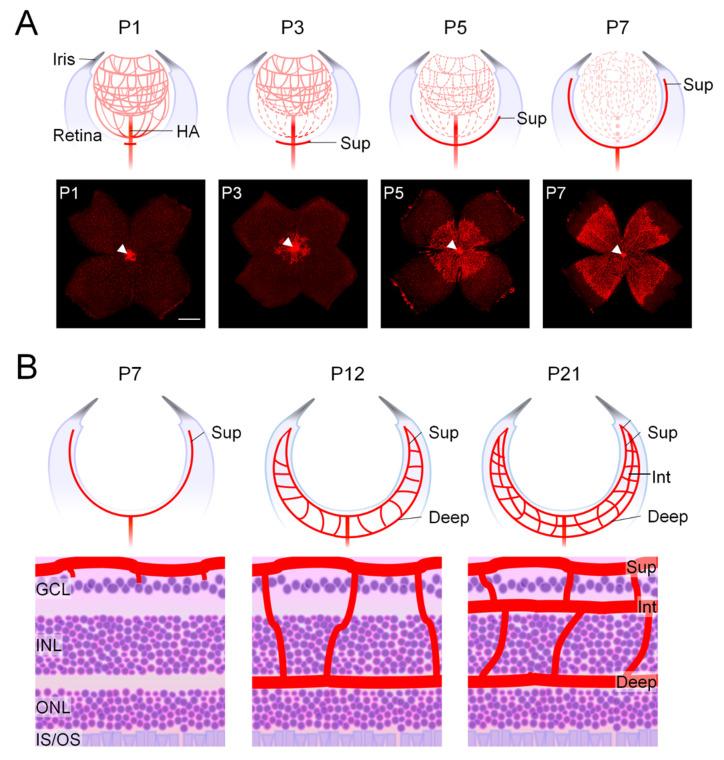
**Development of mouse retina vasculature.** (**A**) The formation of mouse retinal vessels takes place after birth. The upper panels depict cross-section diagrams of mouse eyes at different stages of development from postnatal day (P) 1 to P7, showing the formation of the superficial retinal vascular plexus (Sup, in red). At the same time, the hyaloid vasculature (in light red) including hyaloid artery (HA), a temporary embryonic blood vessel network supplying the developing eye, regresses. The lower panels provide illustrative photos of the retinal flat mounts from C57BL/6J mice. The blood vessels were labeled with isolectin, resulting in a crimson coloration. At P1, the mouse retina has almost no blood vessels. The superficial vascular plexus emerges from the optic nerve head (white arrowheads) and expands outward in a radial pattern from the central retina. By the end of the first week after birth, it reaches the outer edges of the retina. Scale bar: 1 mm. (**B**) The development of the deep and intermediate vascular plexus in the mouse retina begins around the second week after birth and continues thereafter. The upper panels depict cartoon representations of cross-sections of the eye, providing an overview of the organization of three nuclear layers in the retina (at P7, P12, and P21) throughout the development of superficial, intermediate, and deep vascular layers. The lower panels depict schematic pictures of retinal cross-sections illustrating the maturation process of the three retinal vascular plexuses, highlighted in red. Within the initial week after birth, the superficial plexus extends towards the periphery of the retina. Starting from the second week, the superficial vascular plexus begins to expand vertically into the retina, creating the deep vascular plexus between the inner nuclear layer (INL) and outer nuclear layer (ONL). This deep vascular plexus reaches full development by P12. The intermediate plexus undergoes further growth and maturation between the ganglion cell layer (GCL) and INL, reaching virtually full development by P21. The retinal vasculature consists of deep retinal arteries, intermediate retinal vessels, and superficial retinal vessels. The layers of the retina include the GCL, INL, inner segment/outer segment (IS/OS) of photoreceptors, and ONL. The illustration was reproduced with permission from Liu et al. [43].

Mouse retinal vasculature development initiates at the optic nerve head and continues radially towards the periphery of the retina, and this process is parallel to the regression of the hyaloid vasculature. In developing embryonic eyes, the hyaloid vasculature serves as a temporary circulatory system, supplying oxygen and nutrients to the primary vitreous, inner retina, and developing lens [47,48,49]. After birth, the hyaloid vasculature begins to regress concurrently with the radial expansion of retinal vessels during the first postnatal week, generating the superficial vascular plexus [49] (Figure 1A). The deep and, subsequently, the intermediate retinal vascular plexuses are formed by sprouts from the superficial vessels growing perpendicularly towards the inner retina starting in the second postnatal week (Figure 1B). Hyaloid vessels are mostly regressed by the end of the third postnatal week when the retinal blood vessels have fully matured [46,50]. The immature retinal vessels and remnant hyaloid arteries in neonatal mice during this time, particularly in the first week, are similar to those of preterm newborns in humans, with the partially avascular retina highly susceptible to oxygen-induced retinopathy [29].

#### 2.1.2. The Neonatal Mouse Model of OIR with Consistent High Oxygen Exposure

Pathological features mimicking ROP were observed in mouse retinas subjected to high levels of oxygen in the 1950s, around the same time as the early research of similar findings in the kitten OIR model [42,51]. Later work in mouse retinas further investigated the impact of different oxygen levels on causing ROP-like diseased ocular features [52,53], providing valuable knowledge that influenced the development of the current standardized OIR models.

**Figure 2:**
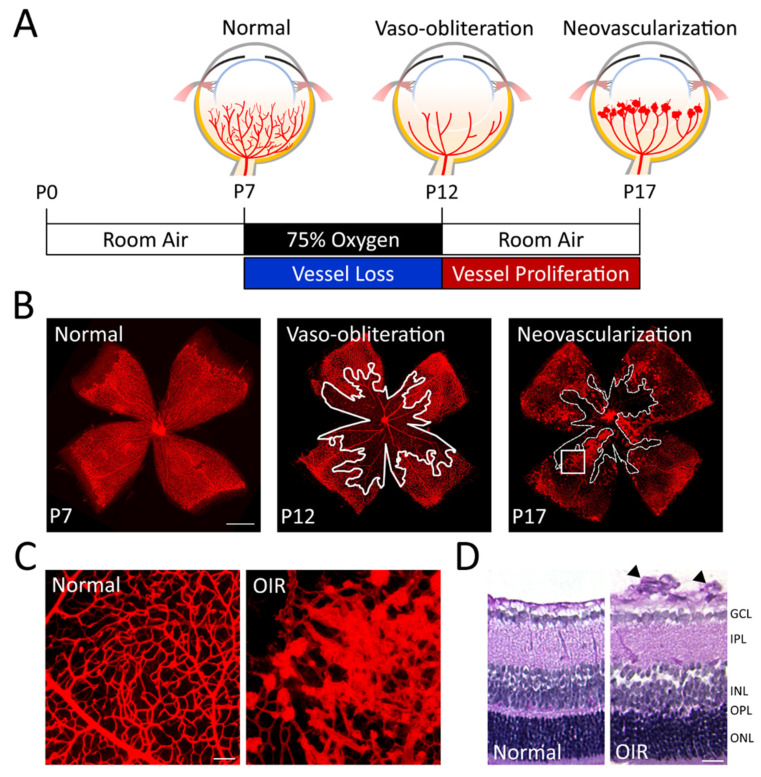
**The mouse model of oxygen-induced retinopathy (OIR) with consistently high oxygen levels.** (**A**) A cartoon illustration depicts the scheme of mouse OIR. Neonatal litters are subjected to 75% oxygen from P7 to P12. Exposure to high levels of oxygen, known as hyperoxia, inhibits the growth of blood vessels in the retina and causes the existing underdeveloped blood vessels to regress, resulting in a central area of vaso-obliteration (VO). At P12, return to normal oxygen levels and hence relative hypoxia causes regrowth of vessels towards the VO zone and abnormal vessel proliferation at the boundary between vascular and avascular zones. NV reaches its peak severity at P17. (**B**) The images display retinal flat mounts stained with isolectin (red) from both normoxic and OIR retinas. The left panel illustrates the normal retinal vasculature at P7. The middle panel demonstrates VO at P12 in OIR, with the area of vessel loss outlined in white. The right panel exhibits pathologic NV at P17 in OIR, with the vessel loss area outlined by a white dashed line. Scale bar: 1 mm. (**C**) Normal blood vessels (left panel) in mice exposed to room air oxygen levels, and abnormal newly formed blood vessels (right panel) in animals with OIR at P17. The image in the right panel was magnified from the rectangular area in (**B**). Scale bar 20 μm. (**D**) The left panel shows cross-sections of normal mouse retinas, whereas the right panel shows cross-sections of OIR retinas at P17. The neovascular tufts (black arrows) in the OIR retinas originate from the superficial retinal vascular layer and extend into the vitreous. The retinal nuclear and vascular layers include the deep retinal vessels, ganglion cell layer (GCL), inner nuclear layer (INL), intermediate retinal vessels, inner plexiform layer (IPL), outer nuclear layer (ONL), outer plexiform layer (OPL), and superficial retinal vessels (Sup). Scale bar 20 μm. The figure was reproduced with permission from Liu et al. [43].

The existing OIR model in mice, as standardized in the 1990s by Smith et al., is commonly used with several advantages, including genetic manipulation in mice, and standardized assessment of retinopathy severity aided by better visualization of retinal vasculature [28,54]. In this model, newborn mice are exposed to 75% ± 2% oxygen for five days beginning at P7 and returned to room air at P12 until the endpoints of P17 (Figure 2A). Between P7 and P12, blood vessels in central retinas partially recede under hyperoxic conditions, which causes VO, resembling the first phase of ROP. At P9, the greatest amount of vessel loss can be observed, and the vaso-obliterated areas slowly revascularize after that [55,56]. At P12 after returning to normal room air, the vaso-obliterated retinal areas develop ischemia and hence become hypoxic. This stimulates hypoxia-induced pro-angiogenic factors, such as VEGF and EPO, and results in both uncontrolled compensatory pathological NV and physiological revascularization of the VO area (Figure 2B), which resembles the second proliferative phase of human ROP [28,57,58,59,60]. Pre-retinal vascular tufts, another term for these diseased neovessels, are disorganized, small-caliber clusters of NV that protrude intravitreally and grow at the junction between vascular and avascular zones (Figure 2C,D). The amount of pathological neovessels reaches its peak severity at P17, the typical endpoint of the mouse OIR model, with disruption of the blood–retinal barrier and hence increased vascular leakage. After P17, abnormal pre-retinal NV begins to regress spontaneously gradually, and almost fully resolves by approximately P25 [54]. OIR mice also exhibit compromised visual function with dampened a- and b-waves amplitudes of full-field ERG in 4-weeks old OIR-exposed mice that gradually recover over time by 6 to 8 weeks; however, the amplitudes of the oscillatory potential (OP) remained low after returning to normal oxygen levels through 8 weeks [61], indicating prolonged suppression of inner retinal function and the chronic nature of OIR’s impact on retinal function.

Overall, the mouse OIR model is broadly used to study the etiology of ROP as a rare pediatric disease, because of its ease of genetic manipulation and its reproducible induction of NV. In addition, OIR is also useful for investigating more prevalent adult vascular eye diseases with less defined or reproducible experimental models. For example, existing animal models of diabetes do not produce the proliferative stage of diabetic retinopathy; hence, OIR is commonly used as a dependable model for the neovascular component of diabetic proliferative retinopathy and other ischemic proliferative retinopathy. Molecular insights obtained from OIR studies are also highly relevant for neovascular age-related macular degeneration and are additionally valuable for studying various disorders in other organs associated with ischemia-induced aberrant NV [62,63]. The widespread usage of the mouse OIR model in ROP and retinopathy research can be attributed to its consistent reproduction of VO and NV. One drawback, however, is that in contrast to the peripheral avascular retinal areas of the human ROP, the area of VO in mouse OIR is in the central retina. In the rat OIR model with cycling oxygen exposure, the spatial localization of the peripheral avascular zone is more accurately represented.

Variations in the mouse angiogenic response and, consequently, OIR susceptibility are strain-dependent [64,65]. 129S strains with a mixed background often exhibit higher levels of neovascular response and are more angiogenic than inbred C57BL/6 strains in OIR; on the other hand, the angiogenic response in the albino BALB/cByJ strain is lower than C57BL/6. Even within the same strain, vendor-related sub-strain variations in OIR neovascularization may occur [29]. Selecting the right mouse strains for OIR studies requires taking these strain factors into account.

The postnatal weight growth of mouse pups is an additional critical factor to consider when assessing the OIR phenotype. The degree of clinical ROP in neonates can be accurately predicted by postnatal weight gain [66,67,68]. Similarly, in the mouse OIR model, the poor postnatal weight gain group (<5 g at P17) exhibits a dampened vascular response and an extended phase of retinopathy compared to pups with medium (5–7.5 g) and extensive (>7.5 g) weight gain [69]. To properly interpret OIR results, mice must be kept within the normal weight range with their weight gain closely monitored. It is best to exclude runty pups (less than 5 g at P17) with inadequate OIR response from data analysis. Other additional factors, such as the effects of hypoxia on breastfeeding mothers [52,70], may affect the parental care of pups and hence their predisposition and the severity of the OIR vascular phenotype. When necessary, surrogate mouse mothers may be considered.

### 2.2. The Rat Model of OIR with Cycling Oxygen Treatment

In 1954, Patz presented the first demonstration of the rat model of ROP as pre-retinal NV occurred when newborn rat pups were exposed to a continuous high oxygen content of 60–80% [13]. Rats exposed to a hyperoxic environment in later research also showed aberrant vaso-proliferation with variable inconsistencies [71,72]. Researchers have studied and optimized human ROP modeling procedures over time for more effective experiment conditions [73,74,75]. Clinical research has uncovered that oxygen variation, in addition to constant oxygen supplementation, raises the risk of severe ROP [76,77]. Premature newborns may have very rapid fluctuations in the partial pressure of dissolved arterial oxygen, which can result in alternating episodes of severe and prolonged hyperoxemia and hypoxemia [76], raising the risk of developing ROP.

**Figure 3:**
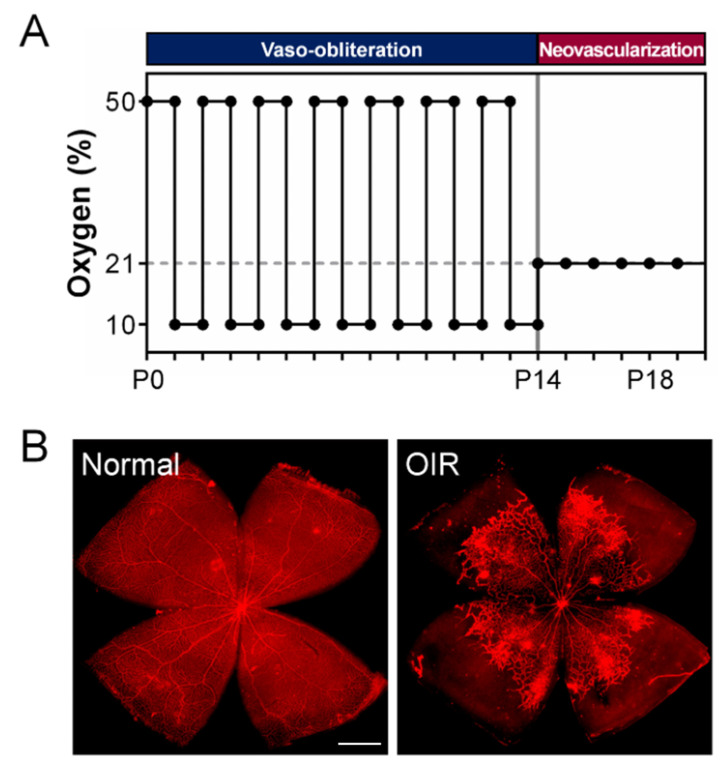
**The rat model of oxygen-induced retinopathy (OIR) with cycling oxygen levels.** (**A**) A schematic illustration of the rat OIR model, namely the 50/10 model. This experimental model subjects the newborn rat litter to alternating 24-h cycles of 50% and 10% oxygen levels from P0 to P14, which delays the development of superficial retinal vessels. Afterward, rat pups are exposed to room air containing 21% oxygen, which causes relative hypoxia and stimulates the growth of new blood vessels (NV). (**B**) Representative retinal flat mount images from normal rats and the 50/10 OIR rat model. Retinas were obtained from P18 normal air control (left panel) and OIR (right panel) rats and stained with isolectin (red) to examine the vasculature. In rat OIR retinas affected, there is delayed growth of blood vessels in the peripheral regions with vaso-obliteration (VO). Pathological NV forms at the border between vascular and avascular areas. Scale bar: 1 mm. Panel B: Image courtesy of Dr. James D. Akula, Boston Children’s Hospital and Harvard Medical School. The figure was reproduced with permission from Liu et al. [43].

Penn and colleagues used these observations to create the rat OIR model with cycling hyperoxia–hypoxia exposure. For the first 14 days after birth, oxygen levels alternate between 50% and 10% every 24 h, after which they are exposed to room air through P20 [8,27,74] (Figure 3). While the oxygen level in the mouse model stays constant during continuous oxygen exposure, the rat model replicates the fluctuation in oxygen tension, which is similar to the fluctuating oxygen levels observed in preterm newborns developing severe ROP [8]. Retinal vascular development is retarded when exposed to cycling oxygen levels, resulting in an avascular zone in the peripheral retina that resembles ROP in humans. At P14, after returning to room air, NV forms at the border between avascular and vascular regions in the mid-peripheral retina.

The rat model of OIR presents clinically important characteristics of ROP—delayed retinal vasculature development and subsequent pathologic NV—as one of its primary advantages. The VO phenotype in the rat OIR model recapitulates the peripheral avascular zone as seen in human ROP [78,79], whereas, in the mouse model, vessel obliteration occurs in the central retinal vessels that are obliterated. In OIR rats, visual function of photoreceptors, bipolar cells and inner retinas were also compromised as reflected by decreased amplitudes of full-field ERG a- and b-waves and OP [80]. Limitations of the rat OIR model include a lack of genetically modified animals, variable and low degrees of NV, and strain-dependent response variance, all of which together limit the use of rats in the evaluation of anti-angiogenic therapy.

### 2.3. Hyperglycemia-Associated Murine Models of ROP

Gluconeogenesis and glycogenolysis help newborns maintain glucose homeostasis in the early postnatal stage of life. It is crucial for the developing central nervous system (CNS). In preterm infants and extremely low-birthweight neonates, a series of factors such as physiological stress caused by surgery, pain, hypoxia, respiratory distress, or sepsis, inadequate insulin secretion, and insulin resistance combined with the newborn’s higher energy consumption might lead to poor blood glucose balance. Hyperglycemia is quite prevalent in very preterm newborns [81]. A blood glucose level between 70 and 150 mg/dL is the ideal goal, with hyperglycemia in neonates characterized by blood glucose levels more than 150 mg/dL [82]. Hyperglycemia at the neonatal stage is a recognized risk factor for ROP [34,35,36,37]. Preterm infants undergoing glucose infusion are frequently susceptible to neonatal hyperglycemia, which is linked to poor suppression of hepatic glucose synthesis following infusion, insufficient pancreatic beta cell response, and insulin insensitivity [83,84,85,86]. Independent of oxygen, hyperglycemia in the first postnatal weeks of life in preterm newborns is strongly linked with the development of proliferative ROP later on [87,88], and slows physiological retinal vascularization [33, 89, 90]. Compared to babies without ROP, neonates with ROP experience hyperglycemia more frequently [90].

Neonatal rats were first used to create a model of hyperglycemia-associated retinopathy by administering a single injection of streptozotocin (STZ) at P1 [32]. Sustained hyperglycemia was quickly produced from P2/3 to P6, retinal angiogenesis was suppressed, accompanied by an inflammatory reaction and subsequent degeneration of inner retinal neurons, all within two weeks of birth. A more recent study created a comparable mouse model for hyperglycemia-associated ROP using injections of STZ every day from P1 to P9 [33]. At P8, hyperglycemia was generated, followed by impeded formation of deep-layer vasculature and photoreceptor dysfunction in the retina. These two hyperglycemia-associated ROP models both replicate the delayed vascular development of ROP, albeit without NV, and could shed light on the processes underlying the retinal vascular damage caused by hyperglycemia in ROP.

### 2.4. Early Feline and Canine Models of OIR

The feline model of OIR is one of the earliest that proved the influence of changing oxygen concentration on the development process of retinal vasculature [13,39]. Ashton and coworkers subjected kittens to 70–80% oxygen for a minimum of four days, inducing retinal vascular obliteration. Afterward, pups were returned to normal air with 21% oxygen, which caused hypoxia-induced vessel proliferation, thus supporting the clinical findings connecting oxygen with ROP [39,91]. The model describes both the vaso-obliterative and the vaso-proliferative phases of ROP and successfully replicates the early stages of the disease. Around the same time as Patz’s group created the first canine model to assess the role of oxygen on puppies’ developing retinal vasculature [42,92], attempts were made to use the canine OIR model for researching ROP. Later studies revised the canine model [40,41,93]. For four days, beagle puppies were exposed to 95–100% oxygen to induce retinopathy. Exposure to hyperoxia delays the development of the retinal vessels, resulting in VO, just like in the kitten model. The vaso-proliferative stage is induced by the relative hypoxia after the puppies are returned to room air [40,94]. Furthermore, the canine OIR model exhibits tractional retinal detachment, a feature seen in the later, more severe stage of human ROP, but not in the feline or murine models. Although there are still certain instances where feline and canine models are utilized, it can be difficult to obtain a statistically meaningful number of animals. Since the 1990s, because of the standardization of murine models and the available genetic modification of mice, the use of OIR models in rats and mice has increased significantly, whereas the feline or canine models have become much less commonly used.

### 2.5. The Zebrafish Model of Hypoxia-Induced Retinopathy

The zebrafish represents an alternate model species for researching angiogenesis-related disorders and medication screening, even though it is not directly relevant to clinical ROP [95]. In adult zebrafish, a model of hypoxia-induced retinopathy was created by using transgenic zebrafish that express stable fluorescent reporters specific to blood vessels [96,97]. By keeping them hypoxic in a tank with 10% air saturation (820 parts per billion) for 3 to 12 days [38,98], new retinal vascular sprouts occurred and reached maximal angiogenic responses by day 12. Later, in zebrafish embryos, a similar model of hypoxia-induced retinopathy was also generated by using chemicals to induce hypoxia and hence stimulating aberrant retinal angiogenesis [38,99].

**Table 1:** A summary of animal models of ROP in different species.

Species	Standardized Experiment Protocol	ROP-like Features	Advantages	Limitation	Reference
**Mouse OIR model**	Two-phase model. **Phase 1:** From P7–P12, 75% ± 2% oxygen for five days. **Phase 2:** Normal room air exposure from P12–P17.	**VO phase**: Regression of blood vessels in central retinas from P8–P12 under hyperoxic conditions. **NV Phase**: Ischemia and hypoxia-induced uncontrolled compensatory pathological NV in mid-peripheral retina from P14–P17.	Mimicking VO and NV in phases 1 and 2 of ROP. Consistent and reproducible NV features. Standardized protocols for quantification of vessel loss, vessel regrowth and pathological NV. Extensive strains with genetic manipulation. Ease of pharmacological interventions.	Spatial location of central VO is in contrast with peripheral VO zone in human ROP. Strain-dependent variations in the angiogenic response. Necessity of surrogate mouse mothers.	[28,54,100]
**Rat OIR model**	Two-phase model. **Phase 1:** From P1–P14, oxygen levels alternate be-tween 50% and 10% every 24 h. **Phase 2:** Normal room air exposure from P14–P20.	**VO phase:** Vessel loss and delayed vascular development in peripheral retinas under hyperoxic conditions from P1–P14. **NV Phase**: Peripheral retinal ischemia and hypoxia stimulates pathological NV starting at P18.	Spatial localization of the peripheral avascular zone is similar to human ROP. Delayed retinal vasculature development. Ease of pharmacological interventions.	Variable and low degrees of NV. Scarcity of genetic manipulation in rats. Strain-dependent response variance.	[27,74]
**Neonatal hyperglycemia associated murine models of ROP**	One-phase rat model. By administering a single injection of streptozotocin (STZ) at P1 in rat model.	**Hyperglycemia** induction from P2/3–P6 **Vascular features**: Delayed development of superficial retinal vasculature at P6 under hyperglycemic conditions.	Clinically relevant neonatal hyperglycemia model with suppressed retinal vascular development. A single injection is sufficient to induce distinct vascular and neuronal phenotype.	Absence of NV phase. Lack of genetic manipulation in rats.	[32]
	One-phase mice model. Administration of STZ from P1–P9 in mice.	**Hyperglycemia** induction from P8–P10 **Vascular features**: Delayed development of deep retinal vascular layer at P10 under hyperglycemic conditions.	Mimics delayed deep retinal vascular development relevant for ROP. Ease of genetic manipulation in mice.	Absence of NV phase. Needs multiple STZ injections to induce hyperglycemia.	[33,101]
**Zebrafish model of hypoxia-induced retinopathy**	By keeping zebrafish in 10% oxygen tank from 3–15 (max) days.	Higher density of vascular sprouts in optic capillary plexus in the hypoxia model.	Highly reproducible retinal vascular sprouts in capillary plexuses.	Absence of vessel loss and regression phase.	[38]
**Feline OIR model**	Two-phase model. **Phase 1:** From P3–P7, 70–80% oxygen for a minimum of 1–4 days. **Phase 2:** Returned to normal air with 21% oxygen from P3–P80.	**VO phase:** complete or partial obliteration of retinal vessels in the VO phase. **NV phase**: revascularization with intravitreal NV.	One of the earliest ROP models with clinically significant retinal vascular features. Also considered a model for ischemic proliferative retinopathy.	Lack of genetically manipulated strain. No retinal detachment. Potential animal ethical concerns. High cost in animal purchase and housing.	[39,102]
**Canine OIR model**	Two-phase model. **Phase 1:** 95% to 100% oxygen for 4 days. **Phase 2:** Returned to room air until P22–P45.	Peripheral VO and NV. Presence of retinal detachment.	Tortuous retinal vessels. Incomplete vascularization of peripheral retina. Persistence of intravitreal NV. Presence of retinal detachment mimicking severe ROP.	Lack of genetically manipulated strain Difficulty in obtaining enough animals. Potential animal ethical concerns. High cost in animal purchase and housing.	[40,103]

### 2.6. Automated Analysis and Quantification of OIR Vascular Features

To aid the visualization of retinal blood vessels during development and pathological vascular features in OIR, several different approaches were developed. The retinal vasculature in mice during development and in OIR can be visualized by fluorescence angiography in vivo followed by retinal flat mounts, which show the lumen of blood vessels. More commonly and effectively, retinal flat mounts can be stained with blood vessel surface markers like isolectin. Key characteristics examined in the OIR rodent models are the retinal areas affected by VO and NV that can be quantified as a percentage of the total retinal area [54,100]. Additionally, cross-sections of the eyes can be performed to illustrate and assess pre-retinal neovascular tufts that protrude into the vitreous cavity [28]. Nevertheless, the measurement of these two crucial OIR features, VO, and NV, is challenging, requiring the involvement of human specialists to interpret the images. Whereas human readers can be expensive, labor- and time-intensive, and prone to bias, computer-aided imaging and analysis software have provided straightforward and standardized quantification of abnormal vascular characteristics [54,100]. A computer-aided semi-quantification approach using ImageJ macros offers notable enhancements in efficiency, strong correlation with existing hand-measurement methods, and consistent and reliable results both within and across individuals, which significantly simplifies the measurement of retinal angiogenesis [100,104].

More recent studies have utilized state-of-the-art machine-learning techniques with deep-learning neural networks to quantify OIR features [105,106]. These networks were trained using a dataset of over 1000 segmentations, with the goal being to achieve complete automation of segmentation in OIR pictures and vascular quantification. A study by Xiao and coauthors established an open-source and fully automated algorithm for OIR analysis using deep learning segmentation [105]. For analysis of VO in OIR, this algorithm obtained correlation coefficients comparable to that seen among experts. Furthermore, this approach outperformed the inter-expert correlation coefficients in quantifying the percentage area of neovascular tufts [105]. In a separate study, Mazzaferri et al. programmed a quantitative retinal vascular assessment (QuRVA) algorithm that utilizes a straightforward machine learning approach and morphological analysis to calculate vascular density and identify abnormal vascular tuft locations in OIR accurately and rapidly, with a significant level of accuracy and consistency compared with human segmentations [106].

While research on OIR animal models frequently focuses on the impact on NV and VO features, another vascular feature, tortuosity, is less frequently emphasized and quantified. Yet, in human ischemic retinopathies, such as ROP, features of elevated levels of vascular tortuosity in arterioles and dilated venules, known as plus disease in ROP, are reliable predictors of the severity of the condition, the necessity for therapy, and the response to treatment. In OIR animals, greater retinal artery tortuosity is exhibited than in normoxia control mice [107]. To quantify vascular tortuosity reliably, a semi-automated analysis method (iROP-Assist) was utilized [108]. This method generates a cumulative tortuosity index as an effective indicator for distinguishing OIR mouse retinas from normoxia controls, where vascular tortuosity correlates with OIR activity [108]. More recently, a deep-learning-based generative adversarial network (GAN) was created to segment blood vessels from retinal OIR flat-mounts, and when combined with iROP-Assist, it produces an automated and accurate assessment of vascular tortuosity [109], allowing accelerated evaluation for ROP and OIR research.

In addition to pathological angiogenesis and vascular tortuosity, the breakdown of the blood–retinal barrier can also be observed in OIR and human retinopathies, and other retinal vascular diseases. Fundus fluorescence angiography (FFA) can be used to visualize vascular permeability and the Miles assay with Evans Blue can be utilized for quantification based on spectrometry [100,110,111,112,113]. Whereas automated quantification of FFA images can pose challenges to the accurate identification of blood leakage from segmenting blood vessels, Comin et al. introduced a new computational technique to pinpoint and measure the extent of retinal vascular leakage based on morphological and log-Gabor quadrature filters, which allowed evaluation of time evolution in FFA in a preclinical model of pathological retinal angiogenesis in very low-density lipoprotein receptor knockout mice [114].

Together, these new developments in automated retinal imaging analysis tools, based largely on deep-learning neural networks, can efficiently measure important vascular features in OIR models and other related ocular vascular disease models. Since most of these programs are open source, these freely available resources can be readily used and modified by researchers to assist their investigative projects.

## 3. Metabolic Regulators Influencing OIR

The balance of numerous pro- and anti-angiogenic factors coordinates the process of angiogenesis in both development and diseases. Providing essential nutrients and growth factors at an early stage facilitates the normal cellular metabolism and development of the retina and retinal vasculature. The animal models of OIR have allowed fundamental investigations of many factors involved in ROP pathogenesis and their associated cellular and molecular mechanisms. With increased recognition of metabolic risk factors in ROP [20], investigation of metabolic regulators may open new ways and strategies for promoting vascular regeneration and countering pathological NV. Here, we focus on hypoxia-dependent growth factors, lipid-derived metabolic regulators, and more recently, factors involved in amino acid metabolism.

### 3.1. Hypoxia, Hypoxia-Induced Growth Factors and VEGF

The discovery of critical roles of hypoxia in retinal angiogenesis and retinopathy has been made possible in part by work in the developing mouse retinas and experimental OIR models. The primary mechanism governing cellular and vascular reactions to hypoxia is the activation of transcription factors known as hypoxia-inducible factors (HIFs) [115]. The development of retinal vasculature depends on HIF-1α activity and is brought on by physiological hypoxia [116]. HIFs regulate many signaling pathways involved in hypoxia, metabolic, and stress response, impacting development, metabolism, inflammation, and vascular response [117], through regulating the transcription of important target genes during low oxygen circumstances [118,119]. Under normoxia, HIF alpha subunits are continuously hydroxylated at proline residues by HIF prolyl-hydroxylase (PHD), allowing the ubiquitination and degradation of HIF. Whereas under hypoxia, HIF PHD, which depends on oxygen as a co-substrate, is inhibited, thereby stabilizing HIF, to mediate target genes, including VEGF upregulation. This makes PHD inhibitors such as dimethyloxalylglycine and roxadustat potentially effective therapies for countering VO and hypoxia-induced NV [120,121]. In addition, targeting HIF regulation itself by inhibiting HIF activation through Topotecan, 2-azahypoxanthine or HIF-1 α siRNA also protected against VO and pathologic angiogenesis, and preserved visual function in the mouse OIR model [122,123,124].

Despite the fact that a single allele deletion of VEGF or its receptors (VEGFRs) was fatal in mice [59,125,126], previous groundbreaking work in the OIR mouse model showed that pathologic retinal NV was induced by VEGF [59] and that retinal NV was suppressed by VEGF inhibition using aflibercept, ranibizumab and bevacizumab (Avastin) in OIR mouse eyes [58,127,128,129,130,131]. Reducing upregulated VEGF though aiming β- adrenergic receptors such as propranolol [132] and isoproterenol [133] also showed some potential in inhibiting NV, although the effects of propranolol in OIR can be inconsistent and strain-dependent [134].

These works, and studies in other animal models of ocular angiogenesis [135,136,137,138], established the experimental basis for current anti-VEGF treatment in age-related macular degeneration and ROP. Reducing the bioactivity of VEGF with low-dose treatment of anti-VEGF therapies was shown to be safe and effective in ROP management [139,140].

In the rat OIR models, retinal VEGFA protein was also considerably higher as opposed to room air [141,142,143]. Overproduction of VEGFA by Müller cells or astrocytes in the retina is considered the culprit for the development of NV in the OIR models [116,144]. Intervention of VEGF inhibitors such as the VEGFA164 antibody [128] and *Vegfa*-shRNA [129] successfully reduced pathological NV in a rat model of OIR. Beyond VEGF, other HIF-responsive angiogenic agents, like EPO [57,60,145] and angiopoietin and Tie2, as well as additional protein-based signaling pathways such as placental growth factor (PlGF), platelet-derived growth factor (PDGF), and fibroblast growth factors, were also extensively investigated and contribute to the pathophysiology in OIR.

### 3.2. Lipid Metabolism and Modulation: Fatty Acid Oxidation, Polyunsaturated Fatty Acids, and Sphingolipids

In addition to growth factors, lipid-derived metabolites play important roles in retinal health and diseases including retinopathies. The retina relies on a range of specific dietary lipids, including retinol and polyunsaturated fatty acids (PUFAs), which include arachidonic (20:4) and docosahexaenoic (22:6) acids (DHA), for appropriate biological functioning, especially in the photoreceptors. While glucose is a primary fuel for retinal neurons through glycolysis [146], transported mainly by GLUT1 [147], GLUT1 deficiency does not impact normal vision despite developmental delay [148], suggesting the possibility of other retinal energy substrates, such as fatty acid oxidation [149].

Being a highly metabolic part of the CNS, the retina depends on both glucose and fatty acid β-oxidation to meet the energy demands of photoreceptor cells [150]. Lipid β-oxidation enzymes such as medium-chain and very long-chain acyl-CoA dehydrogenase, short-chain 3-hydroxy acyl-CoA dehydrogenase (LCHAD), and mitochondrial trifunctional protein (TFP) harboring LCHAD are localized in RPE, photoreceptors, the inner nuclear layer, the ganglion cell layer, and the nerve fiber layer, whereas cytochrome *c* oxidase subunit I are localized in the inner plexiform and nuclear layers [151]. The dysfunctional fatty acid β-oxidation pathway is associated with retinopathy [152] and for children with LCHAD or TFP deficiency, optimum nutritional treatment was linked to the preservation of retinal function and visual acuity, as evidenced by low plasma 3-hydroxyacylcarnitine and high plasma DHA concentrations [153]. Singh (2023) found that fatty acid metabolism is a key metabolic pathway providing OIR resistance to BALB/cByJ compared to the C57BL/6J strain, which is OIR-susceptible. They demonstrated octanoate, a medium-chain fatty acid, as a primary candidate in fatty acid metabolism signature in OIR resistance that could be used to substitute the tricarboxylic acid (TCA) cycle in hyperoxic conditions [154].

Modifications to retinal lipid metabolism significantly impact tissue homeostasis and have been linked to several eye disorders [150,155]. The retina requires high fluidity for proper function, which is provided by omega-3 and omega-6 PUFAs. Omega-3 PUFA is crucial throughout the third trimester and is lacking after preterm birth [30]. Dietary supplementation with omega-3 PUFAs, such as DHA and eicosapentaenoic acid (EPA), decreased retinal obliteration and pathological NV via inhibiting proinflammatory cytokine production in retinal microglia [30], and direct anti-angiogenic effects mediated in part by 5-lipooxygenase metabolite 4-hydroxy-DHA, cytochrome P450 oxidase 2C, and adiponectin [156,157]. Exclusively administering ω-3-PUFAs at the neovascular stage of the OIR mice directly reduces angiogenesis by over 40%, without affecting VO or the regrowth of physiological vessels [158]. In addition to omega-3 and -6 PUFAs, accumulation of a significant quantity of the omega-9 fatty acid mead acid has been reported in OIR, with an increase in the omega-9 pathway as a possible marker of disease severity [159].

Beyond PUFAs, sphingolipids—a class of amphipathic lipids—are also involved in maintaining normal retinal physiology as well as impacted retinal diseases [160]. Bioactive sphingosine 1-phosphate (S1P) is a crucial signaling molecule that regulates vascular endothelial cell functions, including angiogenesis, cell migration, differentiation, survival, and inflammation [161,162,163]. S1P and S1P receptors are linked to pathological retinal NV in addition to normal vascular development. Overexpression of *S1pr1* in ECs inhibits the production of new blood vessels, while the removal of *S1pr1* from ECs exacerbates the formation of neovascular tuft in the OIR retina [164]. S1PR2 is highly activated in ECs under hypoxic stress. *S1pr2* deletion suppresses abnormal blood vessel growth in the vitreous chamber, accompanied by a decrease in EC gaps and reduced infiltration of inflammatory cells in OIR-exposed mice [165]. Production of S1P is catalyzed by the enzyme sphingosine kinase (SPHK). Overexpression of *Sphk1* and *Sphk2* enhances angiogenesis under physiological circumstances [166,167]; yet, in OIR, *Sphk2* lowered the avascular retinal region and aggravated pathological retinal NV [166].

### 3.3. Emerging Role of Amino Acid Metabolism in Angiogenesis and Retinopathy

The significance of EC metabolism in promoting angiogenesis throughout vascular growth and maturation has gained recognition in recent years [168,169]. Blood vessel development in the developing eye enables the transport of nutrients and the elimination of metabolic excess from the neurons of the retinas [29,170]. Sprouting angiogenesis depends on the vascular endothelium’s metabolism of not only glucose and lipids but also amino acids (AAs). Glutamate, asparagine, serine, and glycine are among the AAs that may act as metabolic fuels for multiplying ECs to encourage angiogenesis [171,172,173,174]. Among them, glutamine is the most prevalent non-essential amino acid in the body and may be metabolized to support EC development as well as being an important source of carbon and nitrogen [174,175]. It improves the production of nucleotides and proteins during the growth of ECs and serves as a source of carbon to replenish the TCA cycle important for EC function [171,174]. Glutaminase 1 plays a crucial role in the conversion of glutamine to glutamate. Pharmacologically blocking the activity of glutaminase 1 slows the abnormal growth of blood vessels, whereas glutamine deprivation greatly lowers the proliferation of endothelial cells and the formation of new blood vessels [171]. Glutamine controls not only the balance of redox reactions and the generation of large molecules, but also the formation of new blood vessels by facilitating the manufacture of amino acids through functioning as a precursor for other amino acids like aspartic acid, asparagine, and glutamic acid [176,177]. In deficiency of glutamine, asparagine can be used alternatively as an AA supply to partially restore EC anomalies unique to glutamine shortage [178]. These glutamine and asparagine amino acids were upregulated during hypoxia-induced pathological NV formation in OIR mice retina [179]. To actively move glutamine from the extracellular environment into brain vascular ECs with barrier polarity, sodium-dependent transport systems on the EC abluminal membrane and facilitative transport systems on the EC luminal membrane are required [180].

**Figure 4:**
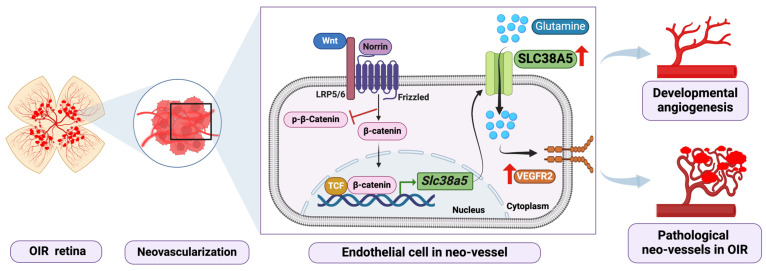
Schematic illustration of a pro-angiogenic role of amino acid (AA) transporter SLC38A5 in oxygen-induced retinopathy (OIR). Pathologic retinal NV (black square) forms when the Wnt receptor and endothelial SLC38A5 expression are both highly expressed in OIR. Wnt/β-catenin signaling is activated in vascular endothelial cells (ECs) by Wnt ligands (Wnts and Norrin), which regulates the transcription of EC-enriched *Slc38a5* potentially through interacting with a TCF-binding site on the promoter of *Slc38a5*. Endothelial SLC38A5 promotes the absorption of AAs by the ECs as an energy source and source of protein synthesis, including glutamine. Increased glutamine and nutrition availability in turn influences VEGFR2 signaling, leading to increased retinal angiogenesis, impacting both developmental angiogenesis and pathologic NV in OIR. Pathologic neovessels in OIR may be suppressed by SLC38A5 deficiency. The figure was adapted and modified with permission from Wang et al. [189]. Created with BioRender.com.

Solute carrier family 38 member 5 (SLC38A5, often referred to as SNAT5: system N sodium-coupled AA transporter 5), is one such transporter for neutral amino acids such as glutamine, asparagine, histidine, serine, glycine, and alanine, to transport them across cell membranes [181]. *Slc38a5* has a significant enrichment in the ECs of the retinal vasculature. Previous research revealed that *Slc38a5* was significantly decreased in both *Lrp5*^−/−^ and *Ndp*^y/−^ retinas, which are experimental models of familial exudative vitreoretinopathy (FEVR) and Norrie disease, respectively, two genetic vascular eye diseases with inadequate retinal vasculature [182,183,184]. Similar retinal vascular defects are present in both retinal disease models due to inherent mutations in Wnt signaling. These include initial delayed or incomplete vascular development, lack of a deep retinal vascular layer, VEGF overproduction in the retina secondary to hypoxia, and abnormal NV in the superficial retinal vasculature [185,186,187,188]. During development, *Slc38a5* is 7–10 times lower in both *Ndp*^y/−^ and *Lrp5*^−/−^ retinas compared with their wild-type controls [182,184], suggesting a possible substantial correlation between *Slc38a5* and the formation of retinal blood vessels. A previous study from our lab investigated the regulatory roles of SLC38A5 in the process of retinal angiogenesis, both in developmental stages and in ischemic retinopathy. We reported that the absence of SLC38A5 in mice significantly slows the growth of blood vessels in the retina and inhibits the abnormal neovascular tufts in mice models of OIR. Blocking SLC38A5 in human retinal microvascular ECs (HRMECs) hinders the development and angiogenic abilities of ECs, reduces the intake of glutamine, and weakens the activity of VEGFR2 [189] (Figure 4). Our results identified SLC38A5 as a novel regulator of ocular angiogenesis by regulating AA availability in retinal ECs.

In addition to glutamine, SLC38A5 may transport other amino acids including serine, asparagine, and glycine. These amino acids have significant metabolic importance in various conditions. The process of serine-glycine metabolism is necessary for angiogenesis and is associated with ECs dysfunction caused by oxidized phospholipids [190]. This transport mechanism may play a part in regulating angiogenesis in eye disorders. For instance, when mice lack the enzyme phosphoglycerate dehydrogenase (PHGDH), which is responsible for synthesizing serine, they experience deadly vascular abnormalities [191]. This same enzyme PHGDH has been genetically linked with macular telangiectasia type 2 (MacTel), a rare eye disease characterized by pathological blood vessel growth within the retina with underlying defective serine biosynthesis [192]. Additionally, Singh et al. (2019) discovered that in the mouse OIR model, the HIF response in retinopathy is influenced by serine and one-carbon metabolism, which is facilitated by the cross-interaction of serine metabolism between the liver and the eye [193]. Increased serine, threonine and glycine metabolism is associated with the mouse model of OIR [20,194].

## 4. Summary and Future Directions

ROP is a global cause of visual impairment and a challenging problem in the field of neonatology. The varying degrees of vision impairment exerted by ROP in preterm infants have a potentially lifelong impact on their vision and a significant influence on the healthcare system and society. Over the past few decades, animal models of ROP have developed in tandem with massive research efforts aimed at improving our comprehension of the pathophysiology of ROP, as well as the processes controlling retinal angiogenesis. The availability of various animal models allowed investigation into the roles of many contributing elements (such as oxygen and hyperglycemia) in ROP pathogenesis. Research on early canine, feline, and murine models of ROP have conclusively established that elevated oxygen levels lead to the obliteration of blood vessels in the developing retina. This established the necessity of carefully monitoring and titrating the administration of exogenous oxygen to preterm infants. Studies in OIR models also provided critical insights into VEGF’s involvement in ROP development, aiding in the development of the current anti-VEGF treatments. In addition to hypoxia-induced growth factors, other less-known molecular pathways are also underlying pathological angiogenesis in retinal ischemia, including fatty acids, and transcriptional and metabolic regulation. Studies of these metabolic regulators may potentially open new windows for identifying alternative strategies to promote vascular regeneration and alleviate the initial VO, thereby preventing the pathological NV phase and subsequent neurovascular damage to the retina. An emerging direction in EC metabolic regulation involves amino acid transport and metabolism which controls the nutrient uptake and homeostasis in ECs and retinopathy. Together, these experimental models of ROP have greatly facilitated not only fundamental research into the underlying cellular and molecular mechanisms but also will be important tools to aid discovery of new ways to protect retinas of not only premature infants but also adults.

## Data Availability

No new data were created or analyzed in this study.
